# Oxyresveratrol Ameliorates Dextran Sulfate Sodium-Induced Colitis in Rats by Suppressing Inflammation

**DOI:** 10.3390/molecules26092630

**Published:** 2021-04-30

**Authors:** Jiah Yeom, Seongho Ma, Jeong-Keun Kim, Young-Hee Lim

**Affiliations:** 1 Department of Integrated Biomedical and Life Sciences, Graduate School, Korea University, Seoul 02841, Korea; intro56@naver.com (J.Y.); aktjdgh8@naver.com (S.M.); 2 Department of Chemical Engineering and Biotechnology, Korea Polytechnic University, Shihung-si 15073, Gyeonggi-do, Korea; kjkim@kpu.ac.kr; 3 School of Biosystems and Biomedical Sciences, College of Health Science, Korea University, Seoul 02841, Korea; 4 Department of Laboratory Medicine, Korea University Guro Hospital, Seoul 08308, Korea

**Keywords:** oxyresveratrol, colitis, anti-inflammatory, intestinal mucus layer

## Abstract

Colitis causes destruction of the intestinal mucus layer and increases intestinal inflammation. The use of antioxidants and anti-inflammatory agents derived from natural sources has been recently highlighted as a new approach for the treatment of colitis. Oxyresveratrol (OXY) is an antioxidant known to have various beneficial effects on human health, such as anti-inflammatory, antibacterial activity, and antiviral activity. The aim of this study was to investigate the therapeutic effect of OXY in rats with dextran sulfate sodium (DSS)-induced acute colitis. OXY ameliorated DSS-induced colitis and repaired damaged intestinal mucosa. OXY downregulated the expression of pro-inflammatory cytokine genes (*TNF-*α, *IL-6*, and *IL-1*β) and chemokine gene *MCP-1*, while promoting the production of anti-inflammatory cytokine IL-10. OXY treatment also suppressed inflammation via inhibiting cyclooxygenase-2 (*COX-2*) and inducible nitric oxide synthase (*iNOS*) expression in the colon, as well as the activity of myeloperoxidase (MPO). OXY exhibited anti-apoptotic effects, shifting the Bax/Bcl-2 balance. In conclusion, OXY might improve DSS-induced colitis by restoring the intestinal mucus layer and reducing inflammation within the intestine.

## 1. Introduction

Inflammatory bowel disease (IBD) is an inflammatory condition of the digestive tract, subdivided into two main pathologies, namely ulcerative colitis (UC) and Crohn’s disease (CD). UC is known to affect only the large intestine, while CD can affect the entire gastrointestinal tract [1]. The symptoms of both include severe diarrhea, weight loss, and pain, in parallel to a chronic and relapsing inflammatory state associated with intestinal immune dysregulation and gut epithelial barrier dysfunction [2,3]. A compromised barrier and increased permeability allow for the infiltration of immune cells, such as macrophages and lymphocytes, followed by the release of cytokines, which in turn drive intestinal injury and pathological changes [4]. Although lifestyle factors, including smoking and hygiene, genetic factors, and the presence of certain bacteria and viruses are known to contribute to IBD, the exact triggers of IBD remain unclear. A number of therapeutics, including anti-inflammatory drugs, and antibiotics, are currently used to treat IBD. However, these only benefit certain patient subgroups and often lead to undesirable side effects [5]. Therefore, further investigation for the identification of alternative therapeutic agents is necessary.

Stilbenoid oxyresveratrol (OXY) is a major component of *Morus alba* L. (mulberry) and is known as a typical antioxidant agent [6]. Antioxidants improve IBD via the suppression of oxidative stress [7,8,9], since reactive oxygen species (ROS) and reactive nitrogen species (RNS) are upregulated in IBD, while the antioxidant capacity of patients is reduced [7,10]. A naturally occurring polyphenol, resveratrol is commonly studied for the treatment of various diseases due to its anti-inflammatory and antioxidant activities [11]. Further, antioxidants quercetin and curcumin were also shown to improve colitis by suppressing inflammation [12,13].

In the intestinal lumen, mucins are formed and secreted by goblet cells. Secreted mucins are contributed to form mucus layer that acts as a barrier between the luminal contents and the epithelial surface. Tight junctions that are composed of transmembrane proteins such as occludin, claudins, and junction adhesion molecule, function to seal the paracellular space by holding cells together. Therefore, enhancing mucin biosynthesis and tight junction integrity is essential for resolving IBD [14]. In vitro, OXY was reported to stimulate mucin 2 (MUC2) production in goblet cells [15] and to promote tight junctions in Caco-2 cells [16]. Although the therapeutic effect of OXY on colitis has not yet been explored, *Ramulus mori* extract containing OXY effectively attenuated colitis by protecting mucosal function in a mouse model of dextran sulfate sodium (DSS)-induced colitis [17]. Besides OXY, *Ramulus mori* ethanol extract contained various components such as resveratrol, mulberroside A, and quercetin. Thus, it was not clear whether the improvement effect of *Ramulus mori* extract on DSS-induced colitis via the regeneration of intestinal mucus layer was derived from OXY alone. In addition, the reference focused on the regeneration of intestinal mucus layer in the DSS-induced colitis mouse model. In this study, we investigated the protective effects of OXY in a rat model of DSS-induced colitis, with a focus on its anti-inflammatory properties and restoring colonic structure. Overall, OXY administration protected the intestinal epithelium via the suppression of inflammation as well as apoptosis.

## 2. Results

### 2.1. Therapeutic Effect of OXY in a DSS-Induced Colitis Rat Model

**Figure 1:**
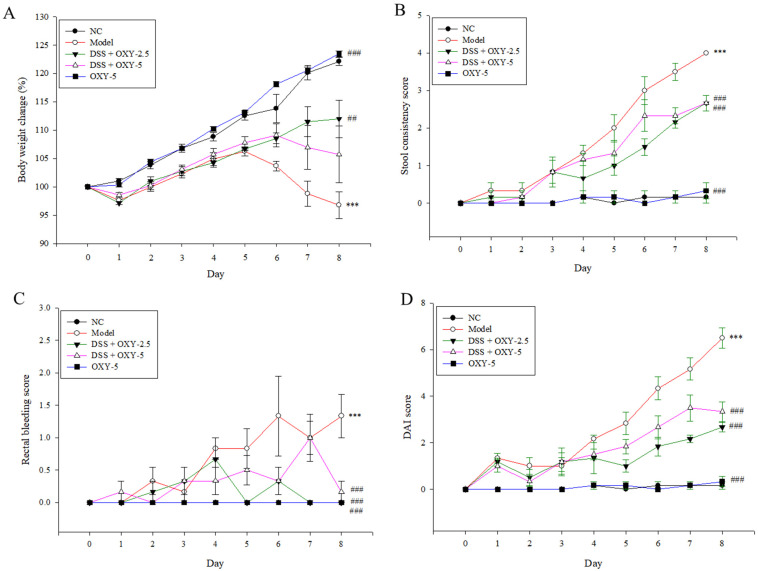
Disease-alleviating effect of oxyresveratrol (OXY) in a dextran sulfate sodium (DSS)-induced colitis rat model (*n* = 8/group). Daily changes of body weight (**A**), stool consistency (**B**), and rectal bleeding (**C**) were monitored, and the disease activity index (DAI) score was calculated by summing scores of these three parameters (**D**). NC, normal control group; model, DSS-induced colitis model group; DSS + OXY-2.5, DSS-induced colitis rats treated with OXY (2.5 mg/kg) group; DSS + OXY-5, DSS-induced colitis rats treated with OXY (5 mg/kg) group; OXY-5, rats treated with OXY (5 mg/kg) alone group. The values are expressed as mean ± SEM. Significant differences among groups were evaluated via ANOVA with Tukey’s post hoc HSD. *** *p* < 0.001 compared with the NC group; ## *p* < 0.01 and ### *p* < 0.001 compared with the model group.

To investigate the therapeutic effect of OXY in a rat model of DSS-induced colitis, several parameters, including body weight loss, stool consistency, and rectal bleeding, were recorded daily after DSS administration in drinking water. Body weight increased for 5 days in the model group. However, after these 5 days, the body weights of model rats significantly decreased compared to the normal controls. While, the administration of OXY significantly inhibited the body weight loss (Figure 1A). After 6 days, body weight in the DSS + OXY-5 (OXY 5 mg/kg-treated) group decreased slightly, but body weight loss was significantly lower than that in the model group. Body weight started to gradually increase after 1 day in the DSS + OXY-2.5 (OXY 2.5 mg/kg-treated) group, following this trend until the end of the experiment. Stool condition became progressively worse in the model group (Figure 1B). The severity of this symptom was significantly ameliorated in OXY-treated rats. Further, while rectal bleeding progressively worsened in model rats 4 days after DSS administration, this was significantly alleviated in the OXY-treated groups at the end of the experiment (Figure 1C). Disease activity index (DAI) scores of the DSS + OXY-treated groups were significantly lower than that of the model group, while no difference was observed between normal controls and the OXY-5 group (Figure 1D). Overall, the DSS + OXY-2.5 group exhibited greater therapeutic benefits than the DSS + OXY-5 group. These results suggest that OXY has colitis-alleviating properties in DSS-treated rats.

### 2.2. Effect of OXY on Colon Length and Spleen Weight in the DSS-induced Colitis Model

**Figure 2:**
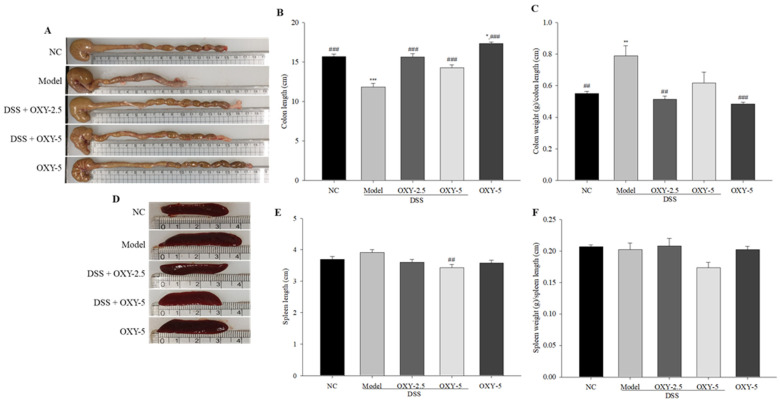
Effect of OXY on the length and weight of the colon and spleen (*n* = 8/group). Colon length was measured (**A**,**B**), after which colon weight was determined and normalized to colon length (**C**). Spleen length was measured (**D**,**E**), after which spleen weight was determined and normalized to spleen length (**F**). NC, normal control group; model, DSS-induced colitis model group; DSS + OXY-2.5, DSS-induced colitis rats treated with OXY (2.5 mg/kg) group; DSS + OXY-5, DSS-induced colitis rats treated with OXY (5 mg/kg) group; OXY-5, rats treated with OXY (5 mg/kg) alone group. The values are expressed as mean ± SEM. Significant differences among groups were evaluated via ANOVA with Tukey’s post hoc HSD. * *p* < 0.05, ** *p* < 0.01 and *** *p* < 0.001 compared with the NC group; ## *p* < 0.01 and ### *p* < 0.001 compared with the model group.

The colons and spleens of rats were collected, and their length (cm) and weight (g) were measured. The colon length of model rats was significantly shorter, while colon weight normalized to colon length was significantly greater compared with normal controls (Figure 2A,B). However, DSS + OXY-2.5 group rats exhibited greater colon length and lower colon weight normalized to colon length relative to model group rats (Figure 2C). Further, the colon length of OXY-5 group rats was greater than that of normal controls. The results indicate that OXY preserves colon length within the normal range in colitis rats.

Although not significant, spleen length in the model group was slightly greater compared to the normal control (Figure 2D,E). Spleen length in the DSS + OXY-5 rats was significantly shorter than in their model group counterparts. While, the spleen weight/length ratio of the DSS + OXY-5 group did not show a significant difference compared to the model group (Figure 2F).

### 2.3. Protective Effect of OXY against the Loss of Colon Surface Epithelium and the Destruction of Goblet Cells in DSS-Induced Colitis

**Figure 3:**
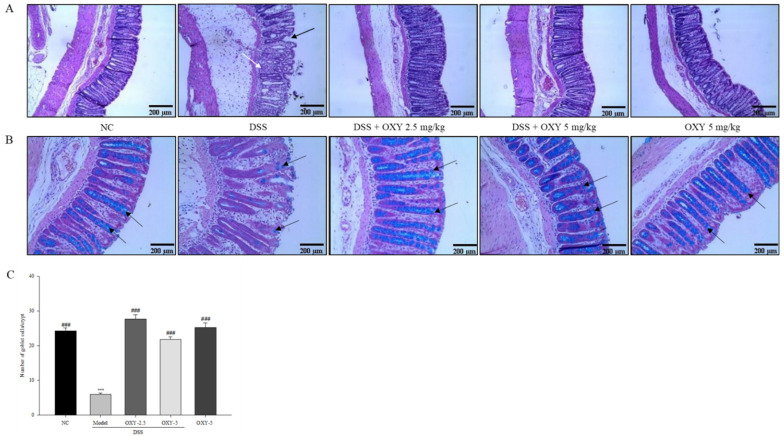
Effect of OXY on histopathological changes in the distal colon (*n* = 8/group). The middle part of distal colons was sectioned and stained with hematoxylin and eosin (H&E) (**A**), infiltrated inflammatory cells (white arrow) and damaged crypt structure (black arrow) as well as with Alcian blue (**B**), goblet cell (black arrow). Goblet cells within a crypt were counted (**C**). NC, normal control group; model, DSS-induced colitis model group; DSS + OXY-2.5, DSS-induced colitis rats treated with OXY (2.5 mg/kg) group; DSS + OXY-5, DSS-induced colitis rats treated with OXY (5 mg/kg) group; OXY-5, rats treated with OXY (5 mg/kg) alone group. The values are expressed as mean ± SEM. Significant differences among groups were evaluated via ANOVA with Tukey’s post hoc HSD. *** *p* < 0.001 compared with the NC group; ### *p* < 0.001 compared with the model group.

To investigate the protective effect of OXY on colon surface epithelium and goblet cells in the DSS-induced colitis model, the middle parts of distal colons from each group were stained with hematoxylin and eosin (H&E), as well as with Alcian blue. The model group exhibited severely inflamed tissue, damaged colon surface epithelium, and distorted crypt structure, when compared to the normal controls (Figure 3A). However, OXY administration resulted in predominantly intact colon histology, with well-preserved surface epithelium and crypt structure compared to the model group. No destruction of colon architecture was observed in the OXY-5 group. In addition, the number of goblet cells per crypt in the model group was considerably lower compared with normal controls (Figure 3B,C). OXY supplementation preserved the number of goblet cells at a similar level to that in the normal control group. The results suggest that OXY protects the colonic structures from DSS-induced damage and preserves the number of mucin-secreting goblet cells.

### 2.4. Effect of OXY on Myeloperoxidase (MPO) Activity in the DSS-Induced Colitis Model

**Figure 4:**
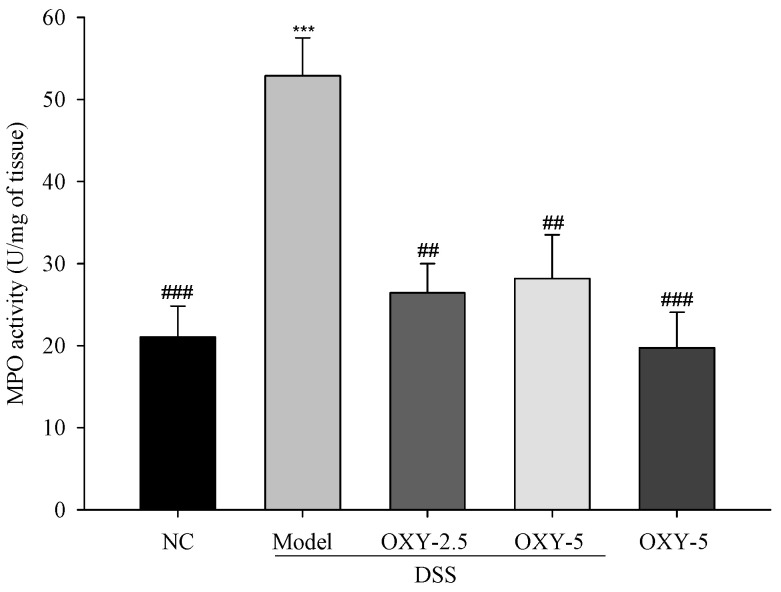
The effect of OXY on myeloperoxidase (MPO) activity (*n* = 8/group). MPO activity in colon tissue was measured and normalized to colon fraction weight. NC, normal control group; model, DSS-induced colitis model group; DSS + OXY-2.5, DSS-induced colitis rats treated with OXY (2.5 mg/kg) group; DSS + OXY-5, DSS-induced colitis rats treated with OXY (5 mg/kg) group; OXY-5, rats treated with OXY (5 mg/kg) alone group. The values are expressed as mean ± SEM. Significant differences among groups were evaluated via ANOVA with Tukey’s post hoc HSD. *** *p* < 0.001 compared with the NC group; ## *p* < 0.01 and ### *p* < 0.001 compared with the model group.

MPO is an enzyme contained within the granules of neutrophils, and increased MPO activity is associated with severe colitis. Thus, MPO activity is used as a marker of inflammation. MPO activity within the colon of model group rats was significantly increased compared to that in normal controls (Figure 4). However, MPO activity in OXY-treated rats was significantly lower relative to their model group counterparts. These results further support the colitis-alleviating effect of OXY.

### 2.5. Effect of OXY on Pro-Inflammatory Cytokine Expression in DSS-Induced Colitis

**Figure 5:**
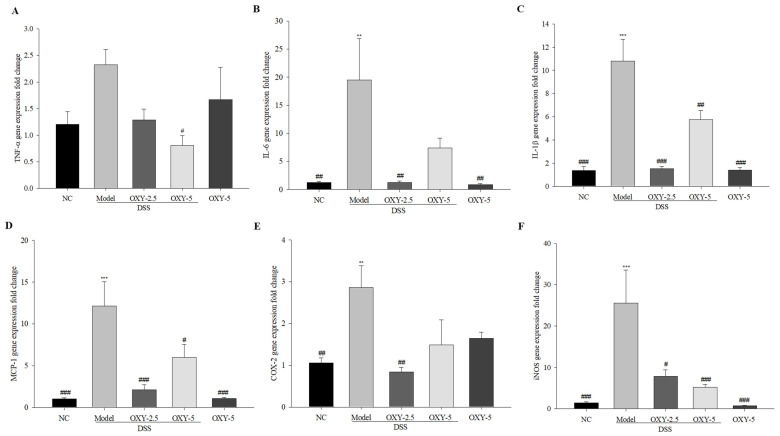
Effect of OXY on the gene expression of pro-inflammatory cytokines and enzymes (*n* = 8/group). The mRNA expression levels of pro-inflammatory cytokines and enzymes were determined via quantitative real-time polymerase chain reaction (qPCR) (**A**‒**F**). NC, normal control group; model, DSS-induced colitis model group; DSS + OXY-2.5, DSS-induced colitis rats treated with OXY (2.5 mg/kg) group; DSS + OXY-5, DSS-induced colitis rats treated with OXY (5 mg/kg) group; OXY-5, rats treated with OXY (5 mg/kg) alone group. The values are expressed as mean ± SEM. Significant differences among groups were evaluated via ANOVA with Tukey’s post hoc HSD. ** *p* < 0.01 and *** *p* < 0.001 compared with the NC group; # *p* < 0.05, ## *p* < 0.01, and ### *p* < 0.001 compared with the model group.

To investigate the immunomodulatory effect of OXY on DSS-induced intestinal inflammation, the gene expression levels of pro-inflammatory cytokines (*TNF-*α, *IL-6*, *IL-1*β), chemokine *MCP-1*, and enzymes (*COX-2* and *iNOS*) were determined. Pro-inflammatory cytokine and enzyme gene expression was significantly higher in model group colons compared to those of normal controls, with the exception of *TNF-*α (Figure 5). The expression of all the other genes assessed was significantly lower in the DSS + OXY-2.5 group compared to the model group. Further, the gene expression of all pro-inflammatory factors, except *IL-6* and *COX-2*, was downregulated in the DSS + OXY-5 group compared to the model group. The OXY-5 group exhibited similar expression levels to those observed in normal controls. These results suggest that OXY suppresses the enhanced pro-inflammatory gene expression in DSS-induced colitis, highlighting its anti-inflammatory effect.

### 2.6. Effect of OXY on IL-10 Production in DSS-Induced Colitis

**Figure 6:**
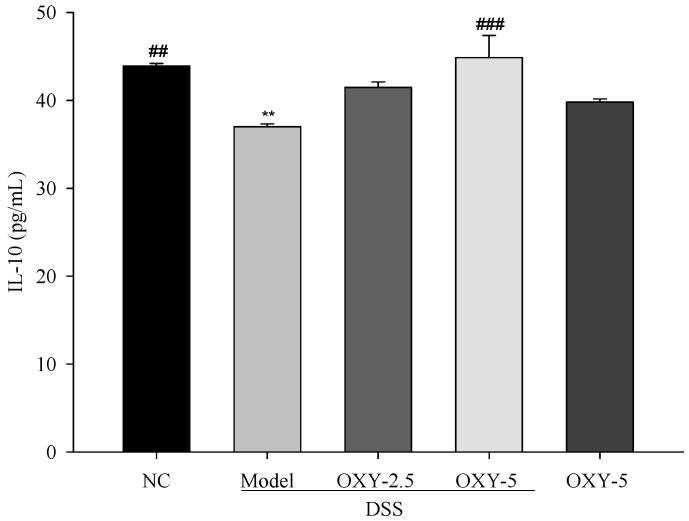
Effect of OXY on IL-10 levels in serum (*n* = 8/group). Anti-inflammatory cytokine IL-10 levels in serum samples were determined via enzyme-linked immunosorbent assay (ELISA). NC, normal control group; model, DSS-induced colitis model group; DSS + OXY-2.5, DSS-induced colitis rats treated with OXY (2.5 mg/kg) group; DSS + OXY-5, DSS-induced colitis rats treated with OXY (5 mg/kg) group; OXY-5, rats treated with OXY (5 mg/kg) alone group. The values are expressed as mean ± SEM. Significant differences among groups were evaluated via ANOVA with Tukey’s post hoc HSD. ** *p* < 0.01 compared with the NC group; ## *p* < 0.01 and ### *p* < 0.001 compared with the model group.

Further, the production of IL-10, a typical anti-inflammatory cytokine, was determined via enzyme-linked immunosorbent assay (ELISA). Serum IL-10 levels of model group were significantly lower compared to those in the normal control (Figure 6). However, DSS + OXY-5 rats exhibited significantly increased IL-10 production compared to the model group. Although the level of IL-10 in DSS + OXY-2.5 rats was also higher than in the model group, the difference was not significant. The OXY-5 group exhibited similar level of IL-10 to that observed in the normal control. The results suggest that OXY might suppress inflammation by enhancing the production of IL-10.

### 2.7. Effect of OXY on Bax and Bcl-2 Expression in DSS-Induced Colitis

**Figure 7:**
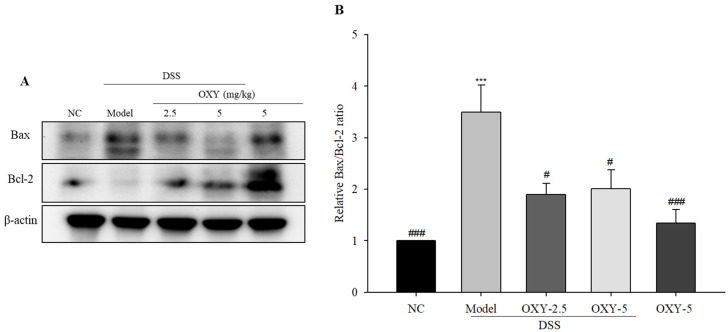
Effect of OXY on the protein expression of Bax/Bcl-2 (*n* = 8/group). Bax and Bcl-2 protein expression was determined via western blot (**A**) and quantified (**B**). NC, normal control group; model, DSS-induced colitis model group; DSS + OXY-2.5, DSS-induced colitis rats treated with OXY (2.5 mg/kg) group; DSS + OXY-5, DSS-induced colitis rats treated with OXY (5 mg/kg) group; OXY-5, rats treated with OXY (5 mg/kg) alone group. The values are expressed as mean ± SEM. Significant differences among groups were evaluated via ANOVA with Tukey’s post hoc HSD. *** *p* < 0.001 compared with the NC group; # *p* < 0.05 and ### *p* < 0.001 compared with the model group.

To investigate anti-apoptotic effect of OXY in DSS-induced colitis, the protein expression of Bax and Bcl-2 in colon tissue were determined via western blot. The model group exhibited significantly upregulated Bax expression and lower Bcl-2 expression, resulting in a significant increase of the Bax/Bcl-2 ratio when compared to the normal control group (Figure 7). OXY-treated groups had significantly lower Bax/Bcl-2 expression ratios compared with the model group. There was no difference between the OXY-5 group and normal controls. Taken together, these results suggest that OXY had an anti-apoptotic effect in the DSS-induced acute colitis model.

## 3. Discussion

OXY is a hydroxystilbene well known for its potent antioxidant activity, and a previous in vitro experiment revealed that OXY had anti-inflammatory effects in LPS-induced inflammation [18]. In this study, we demonstrated the protective effect of OXY in a rat model of DSS-induced colitis. The DSS-induced colitis model has been widely used for the study of acute as well as chronic colitis, depending on DSS concentration and the duration of administration [19]. In this study, 5% DSS administration for 6 days successfully induced acute colitis in rats. Following DSS administration, model rats exhibited characteristic colitis-associated changes, including body weight loss, increased DAI score, reduced colon length, and damaged crypt architecture. Meanwhile, OXY-treated colitis group rats exhibited significantly alleviated symptoms compared to the model group rats. In particular, the intestinal epithelium was recovered and goblet cell numbers were restored. Unexpectedly, OXY-5 group rats did not exhibit any significant improvements when compared to normal controls. Thus, OXY specifically improved colitis-associated deterioration, with no significant effects on the normal intestine.

The worsening of IBD is associated with an upregulation of various pro-inflammatory cytokines and a downregulation of their anti-inflammatory counterparts [20]. This cytokine imbalance leads to a dysregulation of gut homeostasis, driving inflammatory disease. TNF-α levels are a hallmark index of colitis, particularly for UC [21]. When an antigen passes through the compromised gut barrier, activated macrophages and differentiated T helper 1 cells secrete TNF-α as a response. In turn, increased TNF-α aggravates colitis by activating more macrophages, recruiting neutrophils into the site of inflammation and stimulating edema. Further, the production of other pro-inflammatory cytokines, such as IL-6, IL-1β, and IL-33, is also enhanced as a result of TNF-α production. TNF-α is significantly upregulated in UC patients, and anti-TNF-α therapy is quite effective for UC [21]. Similar to TNF-α, another important cytokine, IL-6, mediates the differentiation of various immune cell types and induces the secretion of acute inflammation-associated proteins [22]. Thus, IL-6 is implicated in both UC and CD as well as in colorectal cancer (CRC). Anti-IL-6 therapy might therefore be effective in the inhibition of CRC development as well as for achieving IBD remission. IL-1β is another pro-inflammatory cytokine secreted within the immune cell-infiltrated lamina propria during colitis [23]. It triggers the initial inflammatory cascade, and is known to activate caspase-1, which in turn induces programmed cell death. Chemokine MCP-1 recruits macrophages and monocytes into the inflamed site. MCP-1-deficient mice exhibit lower IBD severity and a reduced production of pro-inflammatory cytokines [24]. Thus, MCP-1 has a major role in the development of colitis. In contrast, IL-10 is a crucial anti-inflammatory cytokine with multiple immunoregulatory functions, including the downregulation of pro-inflammatory cytokines and co-stimulatory molecule expression on macrophages. In this study, DSS administration upregulated both pro-inflammatory cytokine (TNF-α. IL-6, and IL-1β) and chemokine (MCP-1) expression. However, OXY treatment markedly decreased the mRNA expression of pro-inflammatory cytokines while increasing anti-inflammatory cytokine IL-10 production. The anti-inflammatory effect of OXY is well known in other organs. OXY significantly ameliorates allergic asthma by suppressing T-cell response including down regulation of IL-4, IL-5, and IL-13 expression levels [25]. OXY inhibits IL-1β-induced neuroinflammation by blocking the activation of AKT (AKT serine/threonine kinase) and ERK1/2 (extracellular signal-regulated protein kinase 1/2) in human microglia [26]. The COX-2 enzyme, which plays a major role in prostaglandin synthesis, is not detectable under normal conditions, but its expression increases during inflammation [27]. The same is observed for iNOS, another pro-inflammatory enzyme. OXY treatment suppressed COX-2 and iNOS expression, thus reducing inflammation. Another enzyme biomarker of colitis severity is MPO [28]. Within the intestinal mucosa of colitis patients, neutrophils accumulate at inflamed regions and release MPO. Its proteolytic activity is cytotoxic, thus damaging tissue. Importantly, OXY decreased MPO activity, which confirmed the former’s therapeutic effect in colitis. Taken together, OXY ameliorated DSS-induced colitis by suppressing inflammation.

OXY induces autophagy through endoplasmic reticulum (ER) stress signaling pathway, which stimulates MUC2 synthesis in goblet cells [29]. Epithelial autophagy can protect colitis by maintaining mucin secretion and gut microbial population [30]. These results suggest that OXY ameliorates DSS-induced inflammation by increasing autophagy, preventing the destruction of goblet cells, and increasing mucin synthesis.

An increased apoptosis rate is generally observed within the intestinal epithelium of UC patients [31]. Further, the destruction of colonic epithelia and crypts during colitis is driven by apoptosis. Bax and Bcl-2 are major apoptosis-associated proteins, with Bax having pro-apoptotic effects, while Bcl-2 is considered anti-apoptotic. Thus, the Bax/Bcl-2 ratio is often used as an index of apoptosis within tissue. OXY enhances pro-apoptotic proteins and reduces anti-apoptotic proteins in osteosarcoma cells, which induces apoptosis in the cancer cells through inhibition of the STAT3 signaling pathway [32]. However, in this study, OXY decreased Bax/Bcl-2 ratio, which means that OXY protects intestinal goblet cells from apoptosis. In this study, the model group exhibited significantly higher Bax expression and lower Bcl-2 expression compared with normal controls. OXY treatment decreased Bax expression while upregulating Bcl-2 relative to the model group, thus decreasing the Bax/Bcl-2 ratio in rats with DSS-induced colitis. Taken together, OXY inhibited apoptosis in DSS-treated rats, contributing to the amelioration of colitis. 

## 4. Materials and Methods

### 4.1. Animals and Experimental Design

Male Sprague–Dawley (SD) rats (6 weeks of age), weighing 180‒200 g, were purchased from Koatech (Pyeongtaek, Korea). They were housed under a constant temperature of 24 ± 1 °C and a 12 h light/dark cycle at 55% humidity. Animals had ad libitum access to a laboratory chow diet (Harlan diet 2018S, Koatech) and water. All experimental procedures were approved by the Korea University Institutional Animal Care and Use Committee (Approval No. KUIACUC-2019-0049) and performed in accordance with the Guide for the Care and Use of Laboratory Animals (NIH Publication No. 85-23, 1996). A total of 40 rats were allowed to adapt for 7 days and were then randomly divided into five groups (*n* = 8 per group), including a normal control group (NC), DSS-induced colitis model group (model), DSS-induced colitis rats treated with a low dose of OXY (2.5 mg/kg) group (DSS + OXY-2.5), DSS-induced colitis rats treated with a high dose of OXY (5 mg/kg) group (DSS + OXY-5), and rats treated with OXY (5 mg/kg) alone group (OXY-5). Oxyresveratrol (OXY) was obtained from Sigma (St. Louis, MO, USA). After adaptation, rats received a daily oral injection of each test compound for 8 days, and acute colitis was then induced through the addition of 5% DSS (*w/v*) in their drinking water for the first 6 days, with the exception of the normal control and OXY alone groups.

### 4.2. Measurement of DAI as well as the Length and Weight of Colons and Spleens 

The rats were monitored daily for colitis development by observing changes in body weight, stool consistency, and gross rectal bleeding. DAI was scored based on the parameters outlined in Table 1 [33]. The DAI was calculated as the sum of three parameter scores. On the last day of the experiment, rats were sacrificed, and colons as well as spleens were excised and weighed. The lengths of both organs were measured using a ruler.

### 4.3. Histological Analysis

For histological analysis, colons were sectioned longitudinally, fixed in 10% formalin, and embedded in paraffin. Paraffin blocks were sliced into sections, 5 μm in thickness, and stained with H&E for observing colonic damage as well as with Alcian blue for observing goblet cells within crypts. The slides were viewed under a LEICA ICC50 phase-contrast microscope (Leica microsystems, Wetzlar, Germany).

### 4.4. Measurement of MPO Activity

For the measurement of MPO activity, colon specimens were weighed and homogenized in 4 volumes of assay buffer from the colorimetric MPO activity assay kit (ab105136, Abcam, Cambridge, UK). The supernatant was then collected via centrifugation at 13,000× *g* and 4 °C for 10 min. MPO activity was determined using the MPO activity assay kit according to the manufacturer’s instructions.

### 4.5. Quantitative Real Time Polymerase Chain Reaction (qPCR)

Colon specimens were homogenized in TRIzol reagent (Bioneer, Daejeon, Korea), and total RNA was extracted according to the manufacturer’s instructions. As DSS remained in colon specimens, it had to be cleaned up from the total RNA preparation to prevent interference with reverse transcription. To remove DSS from colon samples, total mRNA was mixed with 8 M LiCl (2:1, *v/v*) and incubated on ice for 2 h [34]. The mixture was then centrifuged at 14,000× *g* and 4 °C for 30 min, after which the supernatant was removed. After repeating the process once again with 200 μL of nuclease-free distilled water (NFDW) and 20 μL of 8 M LiCl, the pellet was precipitated with a mixture consisting of NFDW, sodium acetate, and ethanol at ‒20 °C for 30 min. The pellet was washed with 70% ethanol and dissolved in NFDW. The purified RNA was quantified using a NanoDrop ND-1000 spectrophotometer (Thermo Scientific, Wilmington, DE, USA). RNA was converted to cDNA using a RevertAid First Strand cDNA Synthesis kit (Thermo Fisher Scientific, Waltham, MA, USA). qPCR was performed with a Kapa SYBR Fast qPCR kit (Kapa Biosystems, Woburn, MA, USA) on a StepOnePlus™ Real-time PCR System (Applied Biosystems, Foster City, CA, USA). β-actin was employed as an internal control gene, and the primer sequences used in this study are shown in Table 2. The PCR protocol used was as follows: 95 °C for 10 min, followed by 40 cycles of 95 °C for 15 s, 60 °C for 15 s, and 72 °C for 20 s. Relative expression was determined via the 2^−ΔΔCt^ method (the β-*actin* control was set to 1) [35].

### 4.6. ELISA

Blood was collected from sacrificed rats and subjected to centrifugation at 3000× *g* and 4 °C for 20 min in order to obtain serum. The supernatant was collected, and the levels anti-inflammatory cytokine IL-10 were measured via ELISA using a Quantikine ELISA kit (R&D Systems, Minneapolis, MN, USA) according to the manufacturer’s instructions.

### 4.7. Western Blot

For western blot analysis, 10 mg of colon specimens were homogenized in Pro-Prep protein extraction solution (Intron, Seoul, Korea). The supernatant was collected via centrifugation at 13,000× *g* and 4 °C for 5 min. Total protein concentrations were determined via the Bradford assay (Bio-Rad, Hercules, CA, USA). Equal amounts (10 µg for β-actin; 20 µg for Bax and Bcl-2) of protein from each sample were separated by 10% sodium dodecyl sulfate-polyacrylamide gel electrophoresis (SDS-PAGE). The separated proteins were transferred to a polyvinylidene difluoride (PVDF) membrane (Millipore, Bedford, MA, USA) using a Trans-Blot semi-dry transfer cell (Bio-Rad). The membrane was blocked with 5% non-fat skim milk in Tris-buffered saline (TBS) with 0.1% Tween 20 (TBS-T) at room temperature for 1 h, washed with TBS-T three times, and incubated with the following primary antibodies: anti-Bax (1:500 dilution, sc-7480, Santa Cruz Biotechnology, Santa Cruz, CA, USA), anti-Bcl-2 (1:500 dilution, sc-7382, Santa Cruz Biotechnology), and anti-β-actin (1:5000 dilution, MA5-15739, Thermo Scientific), overnight at 4 °C. The membrane was then washed with TBS-T three times and incubated with a goat anti-mouse IgG (H+L) horseradish peroxidase-conjugated secondary antibody (1:10,000 dilution, NCI1430KR, Thermo Scientific) for 1 h at room temperature. After incubation with the secondary antibody, the membrane was once again washed three times with TBS-T, and antibody binding was detected with the SuperSignal West Femto Maximum Sensitivity Substrate kit (Thermo Fisher Scientific). Blot images were obtained and analyzed using a FluorChem E imaging system (ProteinSimple, San Jose, CA, USA).

### 4.8. Statistical Analysis

All statistical analyses were performed using the Statistical Package for the Social Sciences (SPSS, Chicago, IL, USA) version 24.0. Data are expressed as the mean ± standard error of the mean (SEM). Statistical differences between groups were determined using one-way analysis of variance (ANOVA) followed by Tukey’s post hoc HSD (honestly significant difference) test. A *p*-value of <0.05 was considered statistically significant.

## 5. Conclusions

OXY prevented gut inflammation by regulating the expression of cytokines and inflammation-related enzymes in the DSS-induced colitis rat model, thus decreasing DAI scores, protecting colonic structures, and restoring number of goblet cells from DSS-induced damage. Interestingly, OXY treatment had no significant effect in normal control rats. Taken together, OXY might represent an adjunct therapeutic agent for the prevention and improvement of colitis.

## Figures and Tables

**Table 1:** Scoring system for the calculation of disease activity index (DAI).

Score	Body Weight Loss	Stool Consistency	Bleeding
0	None	Normal	None
1	1–5%	Soft but maintains morphology	A little bloodstain
2	5–10%	Soft	Apparent bloodstain on fecal pellets and rectum +
3	10–20%	Very soft	Apparent bloodstain on fecal pellets and rectum ++
4	>20%	Diarrhea	Gross bleeding

**Table 2:** Primers used for quantitative real-time polymerase chain reaction (qPCR).

Gene	Forward (5′ to 3′)	Reverse (5′ to 3′)
β-*actin*	AGC CAT GTA CGT AGC CAT CC	CTC TCA GCT GTG GTG GTG AA
*TNF-*α	ATG TGG AAC TGG CAG AGG AG	GGC CAT GGA ACT GAT GAG AG
*IL-6*	CCG GAG AGG AGA CTT CAC AG	ACA GTG CAT CAT CGC TGT TC
*IL-1*β	AGG CAG TGT CAC TCA TTG TG	GGA GAG CTT TCA GCT CAC AT
*MCP-1*	ATG CAG TTA ATG CCC CAC TC	TTC CTT ATT GGG GTC AGC AC
*COX-2*	ATC AGG TCA TCG GTG GAG AG	CTC GTC ATC CCA CTC AGG AT
*iNOS*	CAT TGG AAG TGA AGC GTT TC	CAG CTG GGC TGT ACA AAC CT

## Data Availability

The information on the data utilized for analysis is provided in the Methods section of this manuscript.

## Sample Availability

Samples of the compounds are available from the authors.
